# Conjugal violence in Tunisia: the characteristics of marriage

**DOI:** 10.1192/j.eurpsy.2024.1680

**Published:** 2024-08-27

**Authors:** R. Jbir, L. Aribi, I. Chaari, A. Samet, R. Ben jemaa, N. Messedi, J. Aloulou

**Affiliations:** psychiatry B, Hedi chaker hospital university, Sfax, Tunisia

## Abstract

**Introduction:**

Violence is a global phenomenon, destroying the fabric of society and threatening the lives, health and prosperity of all. In recent years, there has been an upsurge in domestic violence in Tunsia. Unfortunately, few studies have focused on the relationship within these couples.

**Objectives:**

To describe the characteristics of marriage between Tunisian couples where domestic violence prevails.

**Methods:**

Our study was descriptive and analytical cross-sectional, carried out with women victims of domestic violence examined in the context of psychiatric expertise.

An anonymous survey was asked to these ladies concerning the socio-demographic characteristics of the wife and spouse and the characteristics of the marriage.

**Results:**

Our population was made up of 122 couples. The average age of ladies was 35.66 years (from18 to 64 years). As for the spouses, their average age was 41.68, with extremes of 22 and 70.

92,6% of couples had at least one child.

Professionally, (6.6%) of the husbands were inactive and 51.6% (n= 63) of couples had an average socio-economic level.

43.4% (n=53) lived in rented houses, 41% (n=50) owned their own homes, 14.8% (n=18) lived in a room with their in-laws and 0.8% (n=1) were homeless.

The average duration of marriage in our study was 11.16 **±** 9.12 years and extremes of 1 and 40 years.Judicial records were found in 28.7% of assailants (n=35).The majority of women surveyed, 92.6% (n=113), were victims of three types of violence at once (verbal, psychological and physical). Sixty-two women (50.8%) were victims of four types of violence simultaneously (verbal, psychological, physical and sexual).Various causes of violence were reported, dominated mainly by claims for money, sexual problems, drunkenness and infidelity, with prevalence rates of 38.5%, 23.8%, 22.1% and 21.3% respectively.The majority of women, 66.4% (n=81), had been assaulted by their spouses during the first year of marriage. Forty-seven ladies (38.5%) were subjected to violence on a daily basis.According to the survey, 86.9% of women have been assaulted at least once before, and 38.7% of them have reported previous assaults to the police.The first person contacted after the violence was the mother, with a percentage of 48.4% (n=59).53.3% of ladies were assaulted during pregnancy, 43% of whom suffered obstetrical complications of varying severity.

**Conclusions:**

According to our results, there is no typical profile of a couple where conjugal violence can reign.

Neither the length of the marriage nor pregnancy prevented the woman from being a victim of domestic violence.

**Disclosure of Interest:**

None Declared

